# Antiadipogenic Effects of Loganic Acid in 3T3-L1 Preadipocytes and Ovariectomized Mice

**DOI:** 10.3390/molecules23071663

**Published:** 2018-07-09

**Authors:** Eunkuk Park, Jeonghyun Kim, Subin Yeo, Gijeong Kim, Eun-Hee Ko, Sang Woo Lee, Wan Yi Li, Chun Whan Choi, Seon-Yong Jeong

**Affiliations:** 1 Department of Medical Genetics, Ajou University School of Medicine, Suwon 16499, Korea; jude0815@hotmail.com (E.P.); danbi37kjh@hanmail.net (J.K.); awesome_sky@naver.com (G.K.); sjwlskf7@naver.com (E.-H.K.); 2 Department of Biomedical Sciences, Ajou University Graduate School of Medicine, Suwon 16499, Korea; snsnans@naver.com; 3 Nine B Company, Daejeon 34121, Korea; 4 International Biological Material Research Center, Korea Research Institute of Bioscience & Biotechnology (KRIBB), Daejeon 34141, Korea; ethnolee@hanmail.net; 5 Institute of Medicinal Plants, Yunnan Academy of Agricultural Sciences, Kunming 650200, China; wyli2012@126.com; 6 Natural Products Research Institute, Gyeonggi Institute of Science & Technology Promotion, Suwon 16229, Korea

**Keywords:** obesity, adipogenesis, *Gentiana lutea* L., loganic acid, 3T3-L1 cell, ovariectomized mouse

## Abstract

Obesity is caused by an excess storage of body fat, resulting from a chronic imbalance between energy intake and expenditure. *Gentiana lutea* L. (GL) root has been reported to reduce lipid accumulation in the aortic wall of diabetic rats. Here, we performed fractionation and isolation of the bioactive constituent(s) that may be responsible for the antiadipogenic effects of the GL root extract. A single compound, loganic acid, was identified as a candidate component in the 30% ethanol extract of GL. Loganic acid treatment significantly decreased the adipocyte differentiation of 3T3-L1 preadipocytes in a dose-dependent manner. The expression of key adipogenesis-related genes such as adiponectin (*Adipoq*), peroxisome proliferator-activated receptor gamma (*Pparg*), lipoprotein lipase (*Lpl*), perilipin1 (*Plin1*), fatty acid binding protein 4 (*Fabp4*), glucose transporter type 4 (*Slc2a4*), CCAAT/enhancer-binding protein alpha (*Cebpa*), and tumor necrosis factor-alpha (*Tnf*) were significantly reduced following treatment with loganic acid. In vivo experiments in an ovariectomy-induced obesity mouse model showed that loganic acid (oral administration with 10 and 50 mg/kg/day) significantly inhibited body weight gain, total fat increase, fatty hepatocyte deposition in the liver, and adipocyte enlargement in the abdominal visceral fat tissues. These results suggest that loganic acid in the GL root extract has antiadipogenic effects in vitro and in vivo. Loganic acid may be beneficial for the prevention and treatment of obesity, particularly in menopausal obese women.

## 1. Introduction

Obesity is a chronic disease that can lead to abnormal or excessive body fat accumulation and is associated with various metabolic syndromes, including the hardening of blood vessels, a high risk of type 2 diabetes, and cardiovascular diseases [1]. Obesity is mostly caused by excessive food intake, insufficient exercise, and genetic susceptibility [2]. Some cases are caused by medications, endocrine disorders, or mental disorder [3]. Changes in the diet and exercise intervention are effective strategies for the prevention and management of obesity. If behavioral approaches are not suitable, pharmacological medications may be considered to reduce appetite or decrease fat absorption [4]. Early pharmacotherapy for the treatment of obesity can effectively reduce body weight and the risk of medical comorbidities [5]. The main pharmacological treatments for obesity are liraglutide (Saxenda and Victoza), bupropion and naltrexone (Contrave), and methamphetamine (Desoxyn). Although numerous effective medications for weight loss are currently available, there are still limitations, including serious adverse effects and questionable long-term safety.

Herbal plants have been widely used as alternative therapies with modern medicine for many diseases because of their lower side effects and suitability for long-term use [6,7,8]. Many studies have suggested that single pharmacological compounds that are isolated from natural plants have positive therapeutic effects on herbal medicines [9,10].The root of *Gentiana lutea* L. (GL) has been used to improve gastrointestinal motility and liver function as a herbal medicine [11]. Additionally, a recent study reported that GL is effective for body weight control in overweight individuals by reducing their daily energy intake [12]. Physiological bioactive compounds that have been isolated from GL have been identified, such as iridoids (loganic acid), secoiridoids (gentiopicroside, sweroside, swertiamarin, and amarogentin), and xanthones (isogentisin) [13]. These phytochemical compounds promoted protective effects against osteoporosis and rheumatoid arthritis in ovariectomized mice and collagen-induced rheumatoid arthritis rats, respectively [14,15]. The GL root has been reported to reduce total cholesterol in the blood and lipid accumulation in the aortic wall in streptozotocin-induced diabetic rats [16]. This previous study suggested the potential anti-obesity effects of GL root.

This study aimed to identify the bioactive constituent(s) responsible for the anti-obesity effect of GL extract. We performed fractionation and isolation of GL root extract and identified a single compound, loganic acid, as a candidate active constituent. We further investigated the effects of loganic acid on adipogenesis in 3T3-L1 preadipocytes and ovariectomy (OVX)-induced obesity model mice.

## 2. Results and Discussion

### 2.1. Loganic Acid was Isolated and Identified from the 30% Ethanol Extract of GL Root

The inhibitory effect of a 30% ethanol extract of GL root on adipocyte differentiation was investigated by the assessment of the mRNA expression of adipogenesis-related genes, and by the assessment of lipid accumulation in 3T3-L1 cells. The treatment of 3T3-L1 cells with GL root extract significantly reduced the mRNA expression of the glucose transporter type 4 (GLUT4) (*Slc2a4*) and lipoprotein lipase (*Lpl*), and effectively reduced oil red O accumulation (Supplementary Figure S1).

**Figure 1:**
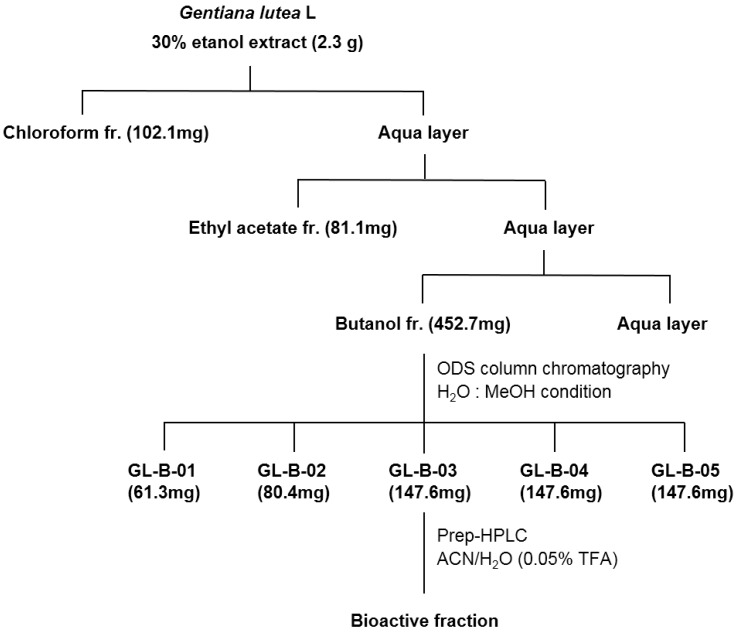
Extraction and fractionation of bioactive components from a 30% ethanol extract of *Gentiana lutea* L. root (GL). The 30% ethanol extract of the GL roots was fractionated using chloroform, ethyl acetate, and butanol. The butanol fraction was further fractionated by column chromatography using octadecylsilyl (ODS) gel and was eluted with methanol in H_2_O (1/4, *v*/*v*) to obtain five fractions (GL-B-01-05). Fraction GL-B-03 was subjected to RP-18 column chromatography and was eluted with acetonitrile (ACN) in H_2_O and 0.05% trifluoroacetic acid (TFA) in a step-gradient manner (10–40%), finally producing a bioactive compound. Abbreviations: fr., fraction; HPLC, high-performance liquid chromatography.

Several previous studies have demonstrated that GL root extract contains various physiologically beneficial bioactive compounds [17,18]. To identify the bioactive constituent(s) that are responsible for the antiadipogenic effects of GL root extract, we performed fractionation of a 30% ethanol extract of GL root (Figure 1).

**Figure 2:**
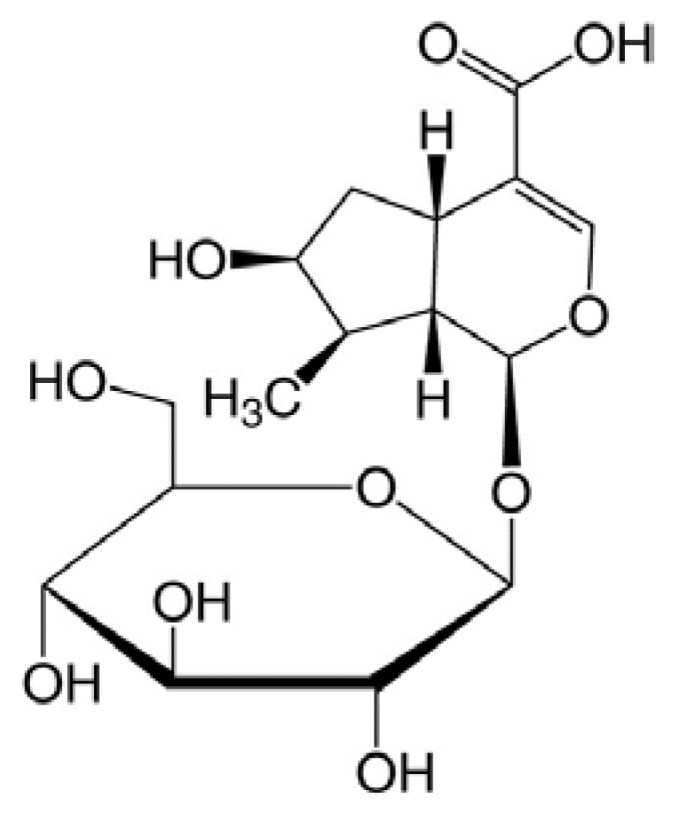
Chemical structure of loganic acid.

The first step of fractionation was carried out using chloroform, ethyl acetate, and butanol as solvents. The ability to inhibit the adipocyte differentiation of 3T3-L1 cells in each fraction was assessed by oil red O staining. The butanol fraction showed reduced oil red O staining of the cells and thus, was further fractionated into five sub-fractions (GL-B-01 to GL-B-05) by octadecylsilyl column chromatography. The sub fractions were assessed for their ability to inhibit adipocyte differentiation by oil red O staining. Because GL-B-03 was the only sub-fraction that was capable of effectively inhibiting adipocyte differentiation in our tested system, we ultimately selected only that fraction for further study. To identify the main constituent of the GL-B-03 sub-fraction, it was subjected to RP-18 column chromatography and was eluted with acetonitrile in H_2_O (0.05% trifluoroacetic acid) in a step-gradient manner (10% to 40%) to produce the bioactive component, which was identified as a single compound, loganic acid, whose molecular formula is C_16_H_24_O_10_. The identification was accomplished by the analysis of 1D and 2D nuclear magnetic resonance and mass spectrometry data, along with a comparison being made with those in the literature [19] (Figure 2).

Loganic acid was previously isolated from GL root as one of the main compounds of GL extract [20]. Various physiological functions of loganic acid have been reported: anti-rheumatoid arthritis effect [14], anti-inflammatory activity [21], neuroprotective effect in Aβ25-35-induced cell death [22], and protective effects on diet-induced hypertriglicerydemia and atherosclerosis [23]. However, the antiadipogenic effect of loganic acid has not been examined to date.

### 2.2. Loganic Acid Decreased Adipocyte Differentiation in 3T3-L1 Cells

We examined the effects of loganic acid on the adipocyte differentiation of 3T3-L1 preadipocytes. Adipocyte differentiation is characterized by the increased expression of adipogenic-related genes, including adipocyte fatty acid-binding protein and lipid-inducing genes [24]. The number of differentiated adipocytes were assessed by oil red O staining to detect lipid accumulation and by the mRNA expression quantification of key transcriptional regulators of adipogenesis-inducing genes, including adiponectin (*Adipoq*), peroxisome proliferator-activated receptor gamma (PPARγ) (*Pparg*), lipoprotein lipase (*Lpl*), perilipin1 (*Plin1*), fatty acid-binding protein 4 (*Fabp4*), GLUT4 (*Slc2a4*), CCAAT/enhancer-binding protein (C/EBP) alpha (*Cebpa*), and tumor necrosis factor (TNF)-alpha (*Tnf*) [25,26].

Adiponectin is mainly produced by fat cells that regulate glucose and lipids [27,28]. Peroxisome proliferator-activated receptor gamma is highly expressed in adipose tissue, the colon, and macrophages, and stimulates lipid synthesis and storage during adipocyte differentiation [29]. Lipoprotein lipase is distributed in adipose, the heart, and skeletal muscle tissue, and mediates the release of fatty acids from circulating lipoproteins for efficient fatty acid uptake and storage [30]. Perilipin1 is associated with lipid metabolism and adipocyte lipolysis during adipocyte differentiation for lipid storage droplet formation in adipocytes [31]. Fatty acid-binding protein 4 is mainly released from adipocytes and is strongly correlated with obesity metabolic diseases as a clinical marker of metabolic and vascular morbidity and mortality [32]. GLUT4 facilitates the transport of glucose that is expressed primarily in adipose tissues, which controls glucose uptake [33]. C/EBP-alpha binds to the leptin promoter and is up-regulated during adipocyte differentiation by inducing the expression of PPARγ [34]. These adipogenesis-related genes play a central role in regulating adipogenesis and lipid metabolism.

**Figure 3:**
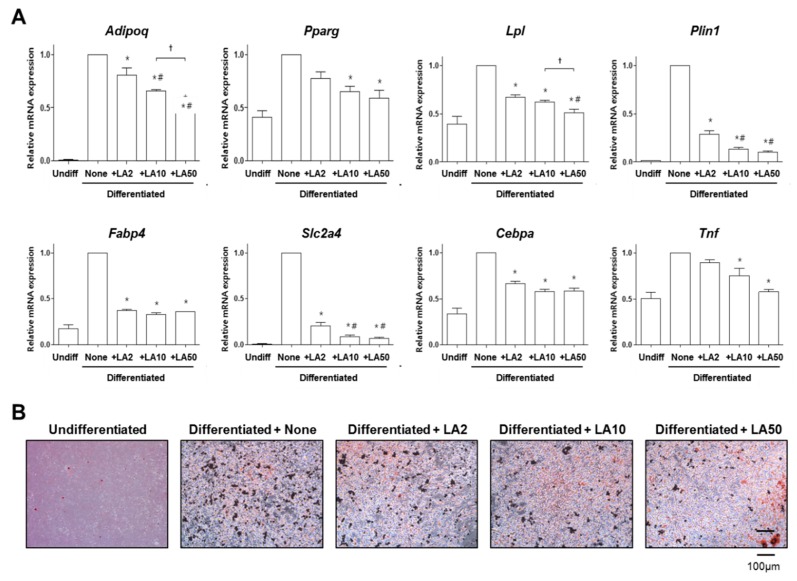
The effects of loganic acid on the mRNA expression of adipogenesis-related genes during adipocyte differentiation of 3T3-L1 cells. The cells were treated with 2, 10, or 50 μg/mL of loganic acid (LA2, LA10, or LA50). (**A**) The expression levels of mRNA of 8 genes were calculated quantitatively by reverse-transcription real-time PCR using gene-specific primers and were then normalized to the *Gapdh* mRNA expression level. *: *p* < 0.05 vs. None, #: *p* < 0.05 vs. LA2, and †: *p* < 0.05 vs. LA10 (Tukey’s HSD post hoc test, ANOVA); (**B**) Lipid accumulation in 3T3-L1 cells was assessed by oil red O staining. Differentiated adipocytes were visualized under a microscope. Abbreviations: Undiff, Undifferentiated; None, non-treated.

Adipocyte differentiation of 3T3-L1 cells was induced by treatment with 0.5 mM 3-isobutyl-1-methylxanthine, 1 mM dexamethasone, and 1 μg/mL insulin [35]. Induction was apparent by the lipid-rich vacuoles that were accumulated in the differentiated adipocytes, which were histologically visualized by oil red O staining [36]. The mRNA expression levels of *Adipoq*, *Pparg*, *Lpl*, *Plin1*, *Fabp4*, *Slc2a4*, *Cebpa*, and *Tnf* were augmented by the induction of adipocyte differentiation (Figure 3A). Loganic acid treatment with 2, 10, and 50 μg/mL did not affect the proliferation of 3T3-L1 preadipocytes during the differentiation period (data not shown). However, loganic acid treatment significantly reduced the mRNA expression of all of the tested adipogenesis-related genes in a dose-dependent manner (Figure 3A). These results indicate that loganic acid prevented adipocyte differentiation of 3T3-L1 preadipocytes by reducing the expression of adipogenic-inducing genes.

Another evaluation method for examining the adipogenesis level is oil red O staining. Positively stained 3T3-L1 cells were augmented by the induction of adipocyte differentiation (Figure 3B). However, loganic acid treatment effectively reduced oil red O-positive 3T3-L1 cells in a dose-dependent manner (Figure 3B).

### 2.3. Loganic Acid Reduced Obesity-Related Phenotypes in Ovariectomy (OVX)-Induced Mice

Based on the results following loganic acid treatment in vitro, we further investigated whether loganic acid influences obesity-related phenotypes in vivo. Since the prevalence of obesity is greater in women than in men [37], as an obesity model animal, we selected OVX-induced obesity model mice. OVX mice are a well-known model of postmenopausal women and are characterized by significantly increased body weight with reduced bone mass and quality [38].

**Figure 4:**
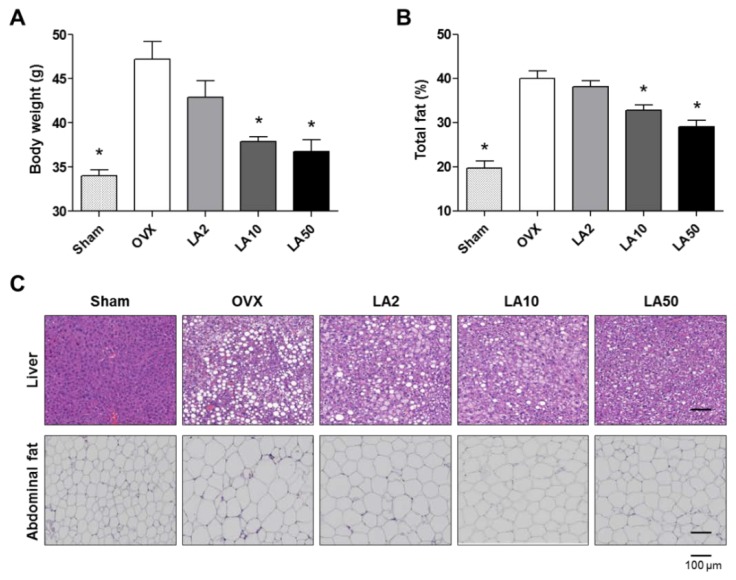
Anti-obesity effects of loganic acid in ovariectomy (OVX)-induced obesity mice. (**A**,**B**) After 12 weeks of feeding, the total body weight and total fat percentage of the mice was measured in the sham-operated mice (Sham), the ovariectomized mice (OVX), and the OVX mice with oral administration of loganic acid (LA2, LA 10, and LA 50: 2, 10, and 50 mg/kg/day, respectively). The body weight and total fat of all of the mice were measured using an electronic scale and PIXImus small animal densitometer, respectively *: *p* < 0.05 vs. OVX mouse group (Tukey’s HSD post hoc test, ANOVA); (**C**) Hematoxylin and eosin (H&E)-stained images of liver and abdominal visceral fat tissues.

Seven-week-old female ddy OVX and sham-operated (Sham) mice were divided into five groups (Sham, *n* = 8; OVX group, *n* = 5; and *n* = 5 in each of three OVX test groups): (1) Sham group; (2) OVX group; (3) test group of OVX mice who were administered 2 mg/kg/day of loganic acid; (4) test group of OVX mice who were administered 10 mg/kg/day of loganic acid; and (5) test group of OVX mice who were administered 50 mg/kg/day of loganic acid. The Sham and OVX control groups were orally administered phosphate-buffered saline and the mice in each test group were orally administered loganic acid for 12 weeks. The average daily food intake for each mouse was as follows: 4.3 g/day for the Sham group, 4.6 g/day for the OVX group, 4.5 g/day for the OVX mice who were administered 2 mg/kg/day of loganic acid, 4.5 g/day for the OVX mice who were administered 10 mg/kg/day of loganic acid, and 4.6 g/day for the OVX mice who were administered 50 mg/kg/day of loganic acid. There were no significant differences in the average daily food intake between the mouse groups who were administered loganic acid and the control OVX group, indicating that loganic acid did not have an influence on the food intake of the mice. At the end of the animal experiment, the body weight and the total fat of all of the mice were measured using an electronic scale and PIXImus small animal densitometer, respectively (Figure 4A,B). Total fat percentage was measured as the total weight of body fat divided by total body weight. As expected, the body weight and total fat percentage were increased in the OVX group compared to in the sham control group. However, the mouse groups who were administered with loganic acid of 10 and 50 mg/kg/day showed significantly lower total body weights and total fat percentages compared to the OVX group. Next, hematoxylin and eosin (H&E)-stained images of liver and abdominal visceral fat tissues of all of the mice were assessed (Figure 4C). Histological images in the OVX group exhibited a high degree of fatty hepatocyte deposition in the liver and adipocyte enlargement in the abdominal visceral fat tissues (Figure 4C). However, the mouse groups who were administered loganic acid at 10 and 50 mg/kg/day presented significantly lower fatty hepatocyte deposition and lower adipocyte size compared to the OVX group (Figure 4C). In addition, the mRNA expression levels of PPARγ (*Pparg*) in the livers and of GLUT4 (*Slc2a4*) and lipoprotein lipase *(Lpl*) in abdominal visceral fat tissues of the mouse groups who were administered loganic acid at 50 mg/kg/day were significantly lower than in those of the control OVX group (Supplementary Figure S2). These results demonstrate the anti-obesity effect of loganic acid in the OVX-induced obesity mouse model.

In this study, we did not test other obesity animal models such as the high-fat diet obesity model. Because we used an OVX-female mouse model for the in vivo experiments to study the anti-obesity effects, it is uncertain whether loganic acid’s anti-obesity effects are non-gender-specific or whether its influence is limited to menopausal obese women.

## 3. Experimental Section

### 3.1. Fractionation, Isolation, and Structure Elucidation of the Bioactive Component

The roots of GL (50 g) were extracted for 24 h at room temperature with 30% ethanol (2 × 0.5 L). After removing the ethanol under vacuum, the aqueous solution was filtered through filter paper. The filtrate was concentrated and the crude extract (2.3 g) was dissolved in 25 mL of H_2_O to form a suspension that was successively partitioned with chloroform, ethyl acetate (EtOAc), and butanol (BuOH) to obtain the chloroform (102.1 mg), EtOAc (81.1 mg), and BuOH (452.7 mg) extracts, respectively. The anti-adipogenetic activities of the 30% ethanol extract and chloroform, EtOAc, BuOH, and water fractions were evaluated by oil red O staining. The BuOH fraction was further fractionated by column chromatography using ODS gel and was eluted with methanol/H_2_O (1/4, *v*/*v*) to give five fractions (GL-B-01 to GL-B-05). Fraction GL-B-03 (147.6 mg) was subjected to RP-18 column chromatography and was eluted with acetonitrile in H_2_O (0.05% trifluoroacetic acid) in a step-gradient manner (10–40%).

### 3.2. Cell Culture and Adipogenesis Induction of Preadipocytes

Mouse 3T3-L1 preadipocytes were cultured in high-glucose Dulbecco’s Modified Eagle’s (DMEM) medium that was supplemented with 10% fetal bovine serum, penicillin (100 U/mL), and streptomycin (100 μg/mL). The differentiation of 3T3-L1 preadipocytes into adipocytes was induced by treatment with 0.5 mM 3-isobutyl-1-methylxanthine, 1 mM dexamethasone, and 1 μg/mL insulin. The cells were co-treated with 2, 10, or 50 μg/mL (molar concentrations were 5.3 μM, 26.5 μM, and 132.8 μM, respectively) of loganic acid. After 3 days, the cells were incubated with 1 μg/mL insulin for another 5 days. The medium containing insulin and loganic acid was changed every 3 days. All of the cultured cells were incubated in a humidified atmosphere at 37 °C under a 5% CO_2_ atmosphere. 

### 3.3. Water-Soluble Tetrazolium Salt (WST) Assay

Mouse 3T3-L1 preadipocytes were incubated in a 96-well plate overnight and were co-treated with different concentrations of loganic acid (2, 10, and 50 μg/mL) in the medium for 48 h. Cell viability was determined in a WST assay. WST solution (20 µL, 5 mg/mL in phosphate-buffered saline, PBS) was added to each well, and the plates were incubated for another 4 h. After removing the media, absorbances were measured at 450 and 655 nm using a microplate reader (BioTek, Winooski, VT, USA).

### 3.4. Oil Red O Staining of Adipocytes

After the adipogenesis induction of 3T3-L1 preadipocytes, lipid accumulation was measured by oil red O staining. Differentiated adipocytes were fixed by 4% paraformaldehyde for 15 min, were washed with PBS three times, and were then stained with oil red O dye for 2 h. The differentiated adipocytes were visualized under a microscope.

### 3.5. Quantitative Reverse-Transcription PCR (qRT-PCR)

Total RNA was extracted from 3T3-L1 cells using TRIzol reagent (Invitrogen, Carlsbad, CA, USA) according to the manufacturer’s instructions (Beckman Coulter, Brea, CA, USA). Total RNA was reverse transcribed using a RevertAid™ H Minus First Strand cDNA Synthesis Kit (Fermentas, Inc., Vilnius, Lithuania) with oligo(dT)_15–18_ and random primers. qRT-PCR was performed with an ABI Prism 7000 Sequence Detection System (Applied Biosystems, Foster City, CA, USA) in a total volume of 25 μL containing 150 ng of cDNA, using a SYBR Green I qPCR kit (TaKaRa, Shiga, Japan).

The specific primers for the adipogenesis-related genes were as follows: 5′-TGT TCC TCT TAA TCC TGC CCA-3′ and 5′-CCA ACC TGC ACA AGT TCC CTT-3′ for mouse *Adipoq*, 5′-GGA AGA CCA CTC GCA TTC CTT-3′ and 5′-GTA ATC AGC AAC CAT TGG GTC A-3′ for mouse *Pparg*, 5′-ATG GAT GGA CGG TAA CGG GAA-3′ and 5′-CCC GAT ACA ACC AGT CTA CTA CA-3′ for mouse *Lpl*, 5′-CAA GCA CCT CTG ACA AGG TTC-3′ and 5′-GTT GGC GGC ATA TTC TGC TG-3′ for mouse *Plin1*, 5′-AAG GTG AAG AGC ATC ATA ACC CT-3′ and 5′-TCA CGC CTT TCA TAA CAC ATT CC-3′ for mouse *Fabp4*, 5′-ACA CTG GTC CTA GCT GTA TTC T-3′ and 5′-CCA GCC ACG TTG CAT TGT A-3′ for mouse *Slc2a4*, 5′-GCG GGA ACG CAA CAA CAT C-3′ and 5′-GTC ACT GGT CAA CTC CAG CAC-3′ for mouse *Cebpa*, 5′-CAG GTT CTG TCC CTT TCA CTC ACT-3′ and 5′-GTT CAG TAG ACA GAA GAG CGT GGT-3′ for mouse *Tnf*, and 5′-TGA CCA CAG TCC ATG CCA TC-3′ and 5′-GAC GGA CAC ATT GGG GGT AG-3′ for mouse *Gapdh*. Relative gene expression levels were quantified and were normalized using *Gapdh*, and the resulting mRNA expression values were expressed as fold-changes compared to the control.

### 3.6. In Vivo Experiments in Obesity Model Mouse

The 8-week-old ovariectomized (OVX, *n* = 25) and sham-operated (Sham, *n* = 8) female ddY mice were purchased from Shizuoka Laboratory Center, Inc. (Hamamatsu, Japan). The mice were maintained on feed (3.0–5.0 g/day) (Feedlab Co., Ltd., Hanam, Korea) and tap water (15 mL/day). All of the mice were housed individually in clear plastic cages under controlled temperature (23 ± 2 °C), humidity (55 ± 5%), and illumination (12 h light/dark cycle) conditions. The mice were orally administered by gavage with different concentrations of loganic acid (2, 10, and 50 mg/kg/day) for 12 weeks. Body weight was calculated using an electronic scale, and total fat was measured using a PIXImus small animal densitometer with on-board PIXI-mus software (GE Lunar, Little Chalfont, UK), and it was adjusted in relation to body weight. The animal research protocol (AMC-133) was approved by the Institutional Animal Care and Use Committee (IACUC) of Ajou University School of Medicine. All of the experiments were conducted according to the institutional guidelines of the committee.

### 3.7. Hematoxylin and Eosin (H&E) Staining

Abdominal visceral fat and liver tissues were collected in cold 4% paraformaldehyde in 0.1 M phosphate buffer at pH 7.4. The fat and liver tissues were post-fixed overnight at 4 °C. Each sample was cut into pieces of 3-μm thickness and was embedded in paraffin for tissue sectioning. The sections were stained with H&E to evaluate the histological changes in the fat and liver tissues.

### 3.8. Statistical Analysis

Statistical analyses were performed using PASW Statistics, version 17.0 (SPSS, Inc., Chicago, IL, USA). The statistical significance of the differences between the groups was calculated by Student’s *t*-test. Multiple groups were compared by a one-way analysis of variance (ANOVA), followed by Tukey’s HSD (honest significant difference) post hoc test for the correction of multiple comparisons. A probability value (*p*) less than 0.05 (*p* < 0.05) was considered statistically significant. Multiple groups were compared by analysis of variance (ANOVA) with corrections for multiple comparisons.

## 4. Conclusions

This is the first study to investigate the antiadipogenic effect of loganic acid isolated from *Gentiana lutea* L. In vitro analysis demonstrated that loganic acid inhibited adipocyte differentiation and reduced the mRNA expression of adipogenic-inducing genes in preadipocyte 3T3-L1 cells. Consistently, in vivo experiments demonstrated that the oral administration of loganic acid in ovariectomy-induced obesity model mice significantly inhibited body weight gain, total fat increase, fatty hepatocyte deposition in the liver, as well as adipocyte enlargement in abdominal visceral fat tissues. These results suggest that loganic acid is a good therapeutic candidate for preventing and treating obesity, particularly in menopausal obese women.

## Supplementary Materials

The Supplementary Materials are available online.
