# Supplementary material for: Antiadipogenic Effects of Loganic Acid in 3T3-L1 Preadipocytes and Ovariectomized Mice
Source: Molecules. 2018 Jul 9;23(7):1663. doi: 10.3390/molecules23071663 (PMC6100558; doi:10.3390/molecules23071663)
Supplement: Supplementary file 1 [file molecules-23-01663-s001.pdf]

Supplementary Figure S1 and S2

Antiadipogenic Effects of Loganic Acid in 3T3-L1 Preadipocytes and Ovariectomized Mice

Eunkuk Park [1,2,3,†], Jeonghyun Kim [1,2,3,†], Subin Yeo [2,3], Gijeong Kim [1,2], Eun-Hee Ko [1,2], Sang Woo Lee [4], Wan Yi Li [5], Chun Whan Choi [6,*] and Seon-Yong Jeong [1,2,3,*]

[1] Department of Medical Genetics, Ajou University School of Medicine, Suwon 16499, Korea; jude0815@hotmail.com (E.P.); danbi37kjh@hanmail.net (J.K.); awesome_sky@naver.com (G.K.); sjwlskf7@naver.com (E.-H.K.)

[2] Department of Biomedical Sciences, Ajou University Graduate School of Medicine, Suwon 16499, Korea; snsnans@naver.com

[3] Nine B Company, Daejeon 34121, Korea

[4] Institute of Bioscience and Biotechnology, International Biological Material Research Center, Korea Research, Daejeon 34141, Korea; ethnolee@hanmail.net

[5] Institute of Medicinal Plants, Yunnan Academy of Agricultural Sciences, Kunming 650200, China; wyli2012@126.com

[6] Natural Products Research Institute, Gyeonggi Institute of Science & Technology Promotion, Suwon 16229, Korea

* Correspondence: jeongsy@ajou.ac.kr (S.-Y.J.); cwchoi78@gmail.com (C.W.C.);
Tel.: +82-31-219-4520 (S.-Y.J.); +82-31-888-6131 (C.W.C.);
Fax: +82-31-219-4521 (S.-Y.J.); +82-31-888-6139 (C.W.C.)

† These authors contributed equally to this work.

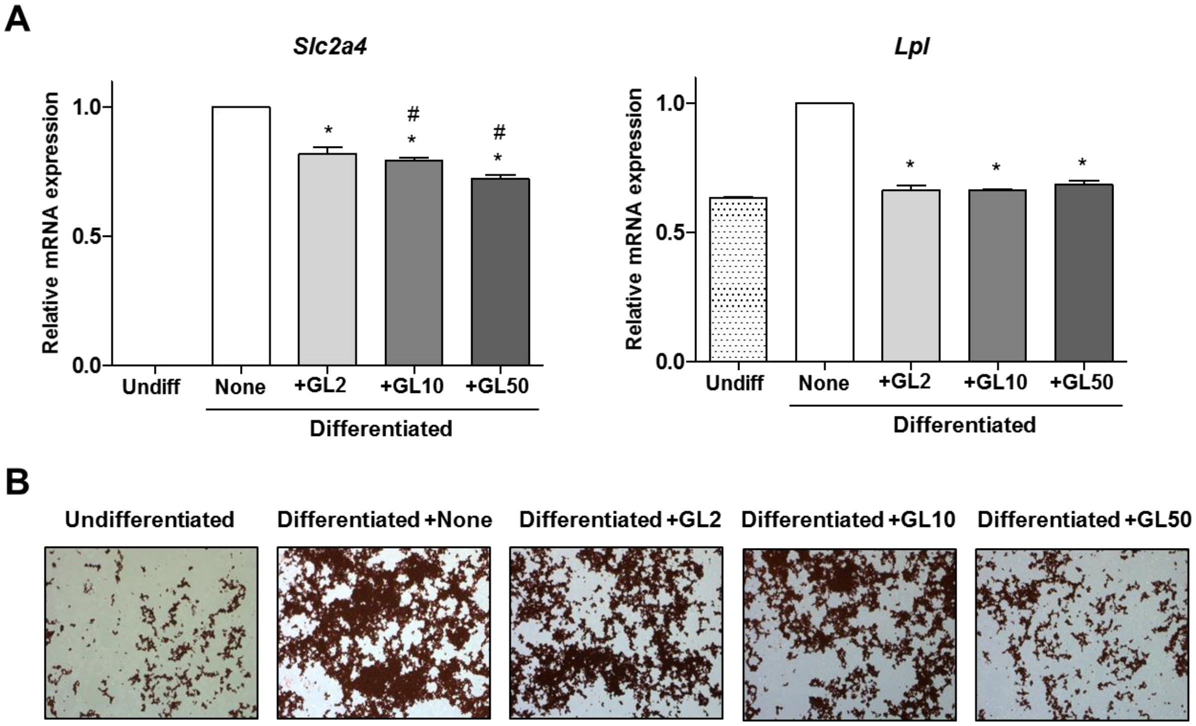

Supplementary Figure S1. Effects of *Gentiana lutea* L. (GL) root on the mRNA expression of adipogenesis-related genes during adipocyte differentiation of 3T3-L1 cells. The cells were treated with 2, 10, or 50 mg/mL of GL (GL2, GL10, or GL50). (**A**) The mRNA expression levels for GLUT4 (*Slc2a4*) and lipoprotein lipase (*Lpl*) were determined quantitatively by reverse-transcription real-time PCR using gene-specific primers and then normalized to the *Gapdh* mRNA expression level. *: $p < 0.05$ vs. None, and #: $p < 0.05$ vs. GL2 (Tukey's HSD post hoc test, ANOVA). (**B**) Lipid accumulation in 3T3-L1 cells was assessed by oil red O staining. Differentiated adipocytes were visualized under a microscope. Abbreviations: Undiff, Undifferentiated; None, non-treated.

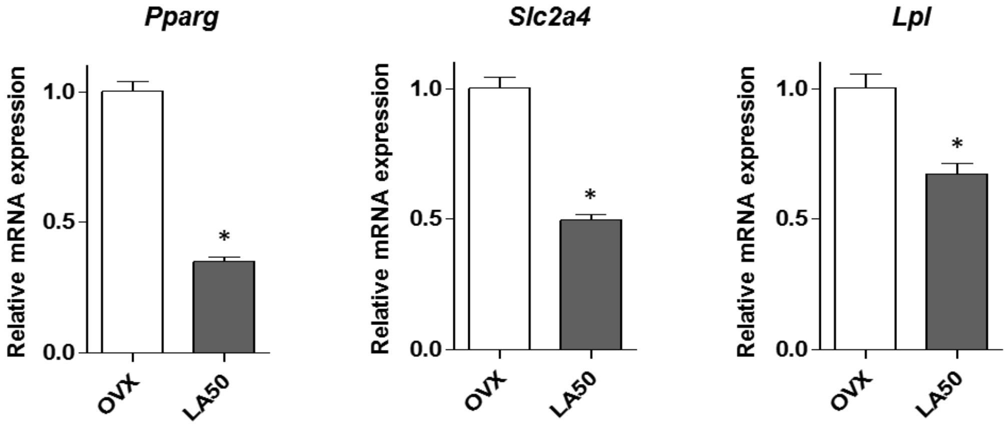

Supplementary Figure S2. Effects of loganic acid on the mRNA expression of adipogenesis-related genes in mice with ovariectomy-induced obesity. After 12 weeks of feeding, total RNAs were extracted from the livers and abdominal visceral fat tissues of ovariectomized mice (OVX) and OVX mice receiving oral administration of loganic acid (LA50: 50 mg/kg/day). The mRNA expression levels of PPARγ (*Pparg*) in the liver, and of GLUT4 (*Slc2a4*) and lipoprotein lipase (*Lpl*) in abdominal visceral fat tissues, were assessed quantitatively by reverse-transcription real-time PCR using gene-specific primers and then normalized to the *Gapdh* mRNA expression level. The resulting mRNA expression values expressed as fold-changes compared to the control. *: $p < 0.05$ vs. OVX (Student's *t*-test).
